# Rare Primary Malignant Melanoma of the Breast Presenting as a Solitary Breast Mass in a 43-Year-Old Male

**DOI:** 10.7759/cureus.48114

**Published:** 2023-11-01

**Authors:** Brittany Q Dang, Brittany Miles, Peter Young, Jing He, Quan D Nguyen

**Affiliations:** 1 Radiology, University of Texas Medical Branch, Galveston, USA; 2 Radiology, Baylor University Medical Center, Dallas, USA; 3 Pathology, University of Texas Medical Branch, Galveston, USA; 4 Radiology, Baylor College of Medicine, Houston, USA

**Keywords:** male breast tumor, melanoma with an unknown primary, rare breast mass, malignant melanoma, advanced melanoma

## Abstract

Primary malignant melanoma of the breast (PMMB) is an extremely rare lesion that carries a poor prognosis. Therefore, it is crucial to examine the patient’s medical history, clinical presentation, and histopathology considering this diagnosis. The rarity of this lesion has made it difficult to identify classic presentations or specific treatment guidelines. Staining for specific biomarkers can be helpful for diagnosis in the absence of melanin pigment on histology. Additional molecular studies to determine gene status can also be useful for targeted immunotherapy and increased survival time for patients. In this paper, we introduce a rare case of PMMB without skin involvement presenting as a solitary breast mass in a male and explore the radiology, histology, evaluation, and treatment options.

## Introduction

Malignant melanoma is a malignant neoplasm of melanocytes that often occurs in the skin but can rarely occur at non-cutaneous sites. It is considered the deadliest form of skin cancer, although highly treatable if detected early. The incidence of melanoma in men has continued to increase over the years and is known to be diagnosed at later stages than in women [1]. Ultraviolet radiation exposure is presumed to be responsible for 60-70% of cutaneous malignant melanomas [2]. Approximately 20% of melanoma cases metastasize to distant sites, such as the liver, lungs, and brain [3].

Malignant melanoma of the breast can manifest as either metastasis to the breast from a primary cutaneous lesion or as primary malignant melanoma of the breast (PMMB) parenchyma. Metastasis of malignant melanoma and other extra-mammary tumors in the breast is considered rare and estimated to account for 1.3-2.7% of all malignant breast cancers, yet it remains the most common primary cause of extra-mammary metastasis to the breast [4,5]. PMMB is the least common variant of melanoma of the breast, making up less than 0.5% of all breast cancers [6].

The majority of metastatic melanomas in the breast are predominantly found in premenopausal women due to their greater amount of breast tissue and regional vascularity [5]. Very few PMMB cases have been reported in men, and any such case of PMMB without cutaneous involvement is considered extremely uncommon. We report a rare case of PMMB without cutaneous involvement in a male.

## Case presentation

**Figure 1:**
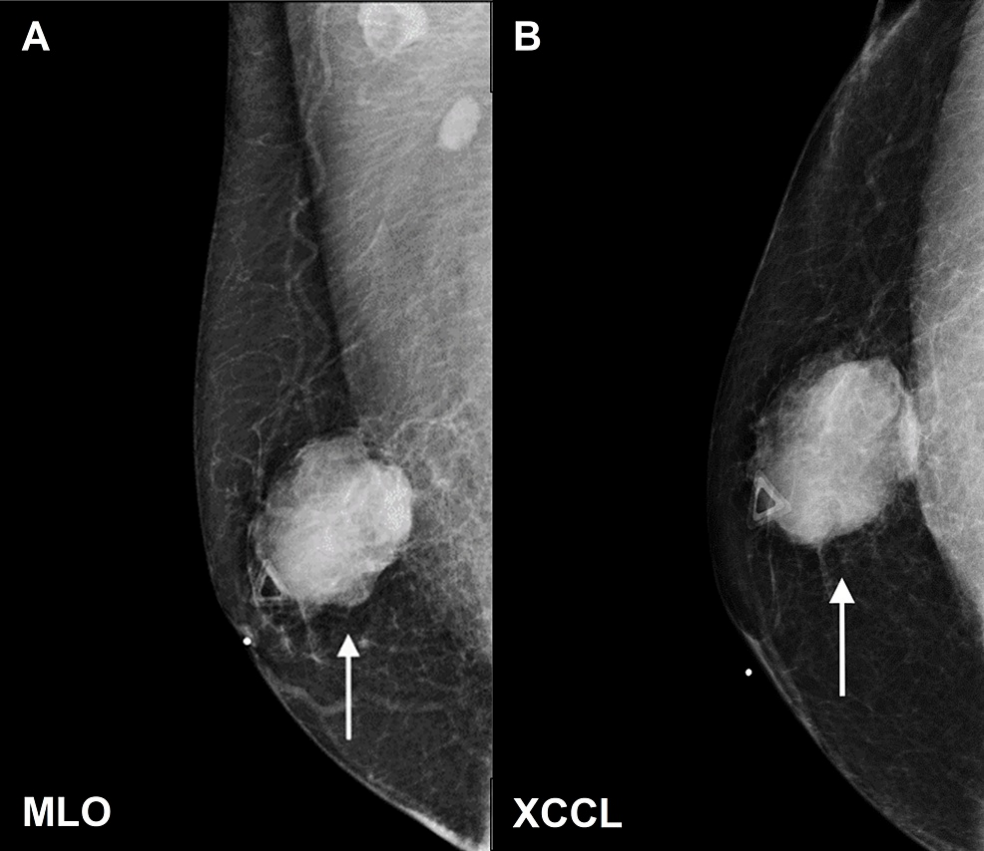
Mammography of the right breast demonstrates a high density and irregular mass with microlobulated margins located at nine o’clock, 3 cm from the nipple. (A) The mediolateral oblique (MLO) and (B) exaggerated craniocaudal view (XCCL) are presented. Two normal axillary lymph nodes are also observed in MLO view that were determined to be benign upon further evaluation with ultrasound and measurement of metabolic activity.

**Figure 2:**
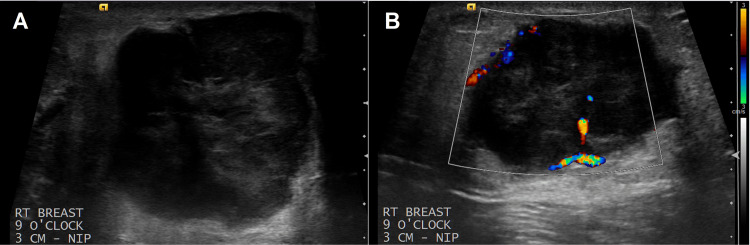
(A) Ultrasonography of the right breast demonstrates a 42 x 32 x 30 mm solid and irregular hypoechoic mass with microlobulated margins at nine o’clock, 3 cm from the nipple. (B) The mass demonstrates increased flow on color Doppler imaging.

A 43-year-old Caucasian male presented with a palpable mass in his right breast that had been increasing in size over the last six months. Physical examination revealed an irregular 4 cm mass in the right breast, which prompted further evaluation with a mammogram. No skin changes were noted upon initial evaluation. He denied other associated symptoms and any previous dark moles, moles that have disappeared, and any family or personal history of melanoma. However, the patient did have an extensive history of occupational ultraviolet radiation exposure. A mammogram showed a high-density irregular mass with a hypoechoic pattern and microlobulated margins on ultrasound in the right breast (Figures 1, 2).

**Figure 3:**
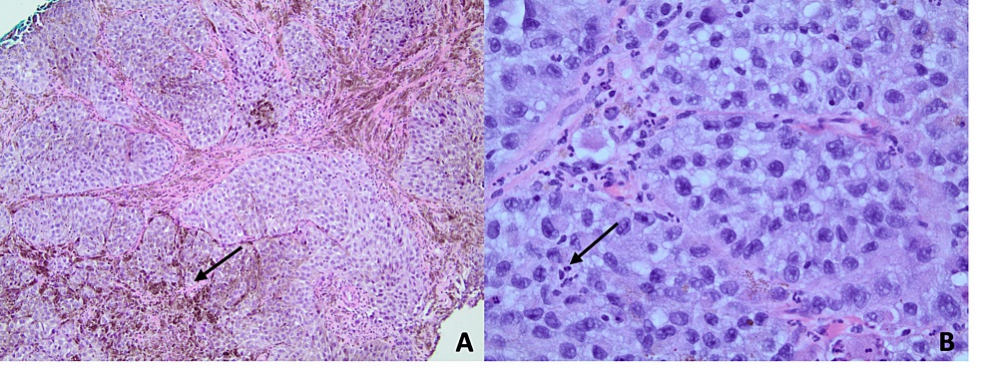
Histological findings of melanoma of the right breast in a male patient. (A) Core needle biopsy of the right breast shows nests of malignant tumor cells in the background of extensive melanin pigment (magnification: 100x). (B) Tumor cells demonstrate epithelioid features with nuclear pleomorphism, irregular nuclear membrane, abundant finely granular cytoplasm, and prominent large eosinophilic nuclei (magnification: 400x).

The suspicious lesion was classified as Breast Imaging Reporting and Data System (BI-RADS) category 4C - high level of suspicion (>50% to <95% likelihood of malignancy), and an ultrasound-guided biopsy was recommended. A core biopsy was performed, and the diagnosis of epithelioid-type malignant melanoma with extensive pigmentation and focal necrosis was made (Figure 3).

A molecular study confirmed the malignancy was v-Raf murine sarcoma viral oncogene homolog B1 (BRAF) positive. A molecular study of this sample showed the tumor to be positive for BRAF mutation p.V600K (GTG>AAG). A fiduciary was placed, and the patient was referred to oncology for treatment and workup for metastatic disease.

**Figure 4:**
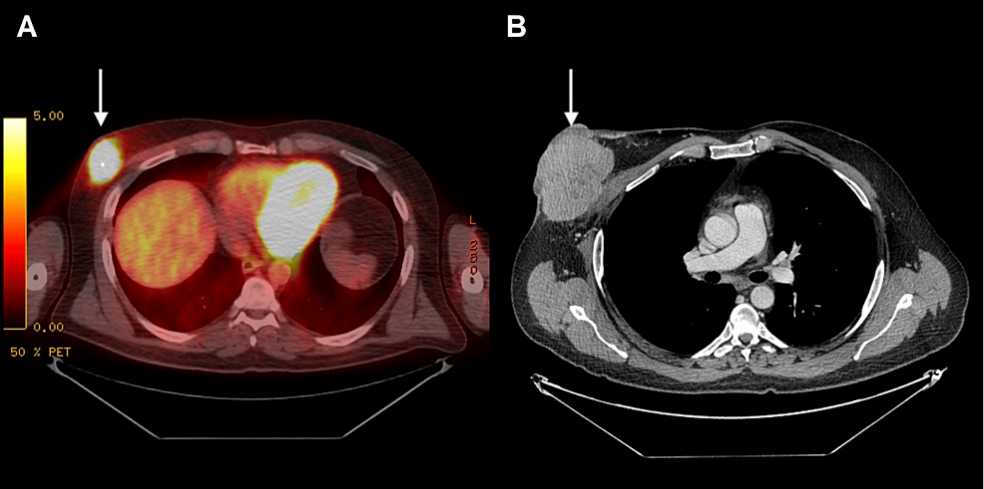
FDG-PET/CT and contrast-enhanced chest CT. (A) FDG-PET/CT shows intense hypermetabolic activity at the lobulated right chest wall mass with an SUV max of 7.4. No FDG-avid nodal or metastatic disease is present. (B) The eight-month follow-up contrast-enhanced chest CT demonstrates a marked increase in the size of the known right chest wall mass. The mass now exhibits areas of internal hypoattenuation and has invaded the pectoralis major. FDG-PET/CT: fluorodeoxyglucose positron emission tomography-computed tomography; SUV: standardized uptake value.

A positron emission tomography-computed tomography (PET-CT) scan revealed that the lesion was fluorodeoxyglucose (FDG)-avid and neoadjuvant therapy with nivolumab was started (Figure 4A). Further studies and exams from dermatology, ophthalmology, and oncology provided no evidence for a primary melanoma source. Surgery to remove the mass would have required a large resection with flap coverage. The treatment options were discussed with the patient, and through shared decision-making, neoadjuvant immunotherapy to decrease the size of the tumor prior to resection was pursued. After eight cycles of nivolumab, repeat studies showed melanoma invasion into the chest wall and surrounding lymph nodes (Figure 4B).

Due to the rapid progression of the melanoma despite neoadjuvant immunotherapy, downgrade of the mass for resection was not achieved, and plans for surgery were further deferred. The addition of a BRAF inhibitor to his neoadjuvant therapy was proposed. Unfortunately, the patient was lost to follow-up and eventually died 20 months after the initial diagnosis of PMMB.

## Discussion

Melanoma of the breast can occur as a result of metastasized primary cutaneous melanoma or PMMB. Metastatic melanomatous lesions of the breast may be asymptomatic or present as palpable, dense, and well-circumscribed nodules on physical examination. Metastasis to the breast commonly appears in the upper outer quadrant as single or multiple circumscribed round masses with slightly irregular margins and various sonographic morphology. Metastatic lesions typically do not present with spiculation, microcalcification, or secondary skin or nipple changes [7]. The typical presentation of PMMB parenchyma is not well-defined due to the limited number of reported cases, which makes distinguishing metastatic disease from primary melanoma difficult when it presents as a solitary breast mass. The current case highlights the rare diagnosis of PMMB in an uncommon population.

Due to the nonspecific radiologic findings, clinical history and histology are critical for diagnosis and treatment. Extensive melanin pigment identified on histology is a diagnostic feature for melanoma. However, amelanotic lesions require further workup with immunohistochemistry (e.g., S100, melanin-A, and HMB-45) to confirm a diagnosis and distinguish melanoma from other malignant tumors [4]. In the current report, the identification of melanin pigment on histology paired with other characteristic histopathological features was diagnostic for melanoma and did not necessitate further immunohistochemistry staining. A molecular study to confirm BRAF V600 status was performed to guide treatment options. Positive BRAF mutation status was also helpful for ruling out clear cell sarcoma of soft parts [8]. The current patient's lack of prior history of melanoma was less concerning for metastatic melanoma. However, regardless of clinical presentation, the presence of melanoma in the breast can indicate extensive disease progression and should prompt full-body evaluation for other metastases and the primary tumor. This may include a thorough skin exam by a dermatologist or further workup by an ophthalmologist to locate any cutaneous or ocular lesions that may be the primary melanoma. Full-body, contrast-enhanced CT may be used to detect the involvement of lymph nodes, surrounding breast tissue, or chest wall [9]. PET-CT can also detect increased FDG uptake, which has been associated with melanoma [10]. Upon evaluation, our patient was found to have a solitary FDG-avid breast mass without other distant metastases or possible primary lesions.

Given the patient’s age at presentation, the finding of a large solitary breast lesion, lack of personal or family history of melanoma, histological findings, and absence of other distant metastases or primary cutaneous lesions, a diagnosis of PMMB was determined. The pathogenesis of PMMB remains poorly understood due to the infrequency of this condition. However, several mechanisms have been proposed, including metastasis to the breast from a regressed primary cutaneous lesion or primary lesion of the breast originating from ectopic melanocytes in the parenchyma.

Following a diagnosis of PMMB, treatment involves wide local resection with clear margins and systemic immunotherapy. In instances where wide local resection with clear margins of a large mass that has spread to local structures and lymph nodes may be challenging, neoadjuvant immunotherapy can be done before surgery to decrease the size of the mass and prevent further progression, such as in this case. However, adjuvant treatment after surgery is more typically recommended when a resectable primary lesion has spread locally. Targeted immunotherapy with BRAF inhibitors, programmed cell death protein 1 (PD-1) inhibitors, and anti-cytotoxic T-lymphocyte antigen 4 (CTLA-4) inhibitors has shown a survival benefit for patients with advanced melanoma [11,12].

Melanoma cancer cells can evade the immune system by upregulating PD-1 ligand, which binds to PD-1 receptors on T cells and suppresses their function. PD-1 inhibitors function by binding to the PD-1 receptor on immune cells, preventing the binding of PD-1 ligand to its receptor. This allows for increased recognition of cancer cells by the immune system and subsequent destruction by T cells. CTLA-4 inhibitors have a similar effect but inhibit a different signaling pathway. CTLA-4 inhibitors bind to CTLA-4 ligand and prevent it from competing with CD28 to bind to B7, which increases the body’s recognition of and activation against cancer cells. BRAF inhibitors function by selectively inhibiting BRAF kinase, which interferes with the proliferation and survival of melanoma cancer cells. However, BRAF inhibitor immunotherapy is reserved for patients with actionable BRAF mutations and is not the first-line treatment even for patients with BRAF mutations. Initial combination therapy with PD-1 inhibitors and CTLA-4 inhibitors has demonstrated greater overall survival in patients with therapy-naive BRAF V600 mutant advanced melanoma compared to initial therapy with BRAF inhibitors and mitogen-activated protein kinase inhibitors [13]. Moreover, current recommendations for patients with advanced melanoma, according to several phase III clinical trials, include first-line treatment with combination therapy of a PD-1 inhibitor (e.g., nivolumab and pembrolizumab) and CTLA-4 inhibitor (e.g., ipilimumab and tremelimumab), regardless of BRAF mutation status to improve overall survival [13,14].

The current patient received several cycles of single-agent therapy with nivolumab, a PD-1 inhibitor, in a neoadjuvant fashion to reduce the size of the metastasis prior to surgical resection but was unsuccessful. Due to the poor prognosis of PMMB, aggressive surgical procedures are often avoided. Historically, the median survival of advanced melanoma has been less than six months [15]; however, with recent combination immunotherapy, this has increased significantly. With combination treatment of nivolumab and ipilimumab or single-agent treatment with nivolumab, the median overall survival time of advanced BRAF-mutant melanoma is estimated to be 72.1 and 45.5 months, respectively [14].

## Conclusions

Although PMMB is extremely rare in men, it is possible and often associated with a poor prognosis. The radiologic appearance of PMMB is nonspecific, making past medical history, clinical presentation, and histopathology crucial in diagnosing PMMB. Additionally, due to the poor prognosis of PMMB, any new finding of a breast mass in a male determined to be melanoma should be systematically investigated, as it may be PMMB or metastatic melanoma that has disseminated beyond the skin or breast. PMMB should be considered when a patient presenting with a solitary melanoma of the breast parenchyma without skin involvement does not have any prior history of melanoma, other distant metastases, or known primary lesion upon full workup. The new development of PMMB in this patient can contribute to the body of knowledge surrounding breast cancer in males.
